# Entangled photon pair generation in an integrated SiC platform

**DOI:** 10.1038/s41377-024-01443-z

**Published:** 2024-05-09

**Authors:** Anouar Rahmouni, Ruixuan Wang, Jingwei Li, Xiao Tang, Thomas Gerrits, Oliver Slattery, Qing Li, Lijun Ma

**Affiliations:** 1 National Institute of Standards and Technology, 100 Bureau Dr, Gaithersburg, MD 20899 USA; 2 Department of Electrical and Computer Engineering, Carnegie Mellon University, Pittsburgh, PA 15213 USA

**Keywords:** Single photons and quantum effects, Silicon photonics

## Abstract

Entanglement plays a vital role in quantum information processing. Owing to its unique material properties, silicon carbide recently emerged as a promising candidate for the scalable implementation of advanced quantum information processing capabilities. To date, however, only entanglement of nuclear spins has been reported in silicon carbide, while an entangled photon source, whether it is based on bulk or chip-scale technologies, has remained elusive. Here, we report the demonstration of an entangled photon source in an integrated silicon carbide platform for the first time. Specifically, strongly correlated photon pairs are efficiently generated at the telecom C-band wavelength through implementing spontaneous four-wave mixing in a compact microring resonator in the 4H-silicon-carbide-on-insulator platform. The maximum coincidence-to-accidental ratio exceeds 600 at a pump power of 0.17 mW, corresponding to a pair generation rate of (9 ± 1) × 10^3^ pairs/s. Energy-time entanglement is created and verified for such signal-idler photon pairs, with the two-photon interference fringes exhibiting a visibility larger than 99%. The heralded single-photon properties are also measured, with the heralded *g*^(2)^(0) on the order of 10^−3^, demonstrating the SiC platform as a prospective fully integrated, complementary metal-oxide-semiconductor compatible single-photon source for quantum applications.

## Abstract (web summary)

Illustration of entangled photon pair generation in an integrated silicon carbide (SiC) platform. This achievement, coupled with SiC’s potential to integrate various electrical, photonic and quantum technologies in the same device platform, offers promises for the scalable implementation of quantum information processing.

## Graphical abstract

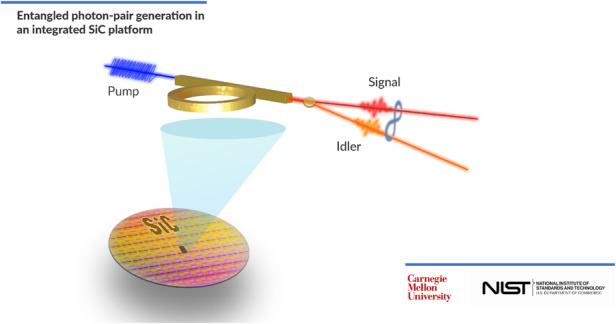

## Introduction

Integrated quantum photonic circuits provide a promising pathway to the scalable implementation of modern quantum technologies. For this purpose, a wide variety of integrated photonic platforms such as silicon-on-insulator and lithium niobate-on-insulator have been actively investigated by the research community^1^. Major building blocks of quantum information processing, including single and entangled photon sources^2–5^, squeezed light^6,7^, efficient photon detectors^8,9^, low-loss waveguides and filters^10^, and quantum memories^11^, have all been demonstrated. These advances greatly benefited crucial applications such as quantum teleportation and photonic quantum computing^12–15^. Despite the impressive progress, significant challenges remain to systematically combine individual components to form complicated quantum systems, which is nowadays typically done through heterogeneous integration due to limited functionalities supported by each material platform^16^.

Recently, silicon carbide (SiC) emerged as a promising photonic and quantum material due to its unique properties^17^. For example, SiC is transparent from visible to the mid-infrared (0.4–5 μm) owing to its large bandgap; the simultaneous presence of second- and third-order optical nonlinearities in SiC underpins a range of nonlinear applications such as second-harmonic generation and Kerr comb generation^18–20^; and the large thermal conductivity of SiC and its robustness make it a preferred material of choice for applications in harsh environments and green technologies. In addition, various color centers with promising quantum properties have been discovered in several polytypes of SiC including 3C, 4H, and 6H^21,22^. These features, coupled with the recent demonstration of low-loss SiC-on-insulator (SiCOI) integrated photonics platform^18,19,23–27^, portend potential disruption of quantum information processing through scalable integration of SiC-based spin defects with a wealth of quantum electrical and photonic technologies on the same chip^17,28^.

Here, we report the demonstration of entangled photon sources in an integrated SiC platform for the first time. Specifically, photon pairs are efficiently generated at the telecom C-band wavelength through implementing spontaneous four-wave mixing (SFWM) in a compact microring resonator in the 4H-SiC-on-insulator platform. With milliwatt-level continuous-wave on-chip pump powers at room temperature, we successfully observed pair generation rates of over 1 million counts per second. The maximum coincidence-to-accidental ratio (CAR) exceeds 600 at a pump power of 0.17 mW, corresponding to an on-chip pair rate of (9 ± 1) × 10^3^ pairs/s. Energy-time entanglement is then created for such signal-idler photon pairs, with the two-photon interference fringe exhibiting a visibility larger than 99% without background subtraction. The heralded single-photon properties are also measured, with the heralded *g*^(2)^(0) on the order of 10^−3^, pointing to strong antibunching. These results demonstrate the viability of the SiC microresonators as promising single- and entangled photon sources, thereby expanding the arsenal available in the SiC integrated platform for a range of chip-scale quantum applications.

## Results

In this work, a compact 43-μm-radius SiC (Norstel AB) microring resonator is employed for photon pair generation in the 1550 nm wavelength band^19^. Given that the Kerr nonlinearity in Norstel 4H-SiC wafers is stronger along the c-axis than the orthogonal directions, our design is focused on the fundamental TM (transverse-magnetic) mode families^29,30^. Linear transmission measurement confirms a free spectral range around 400 GHz for this mode and an intrinsic optical quality factor above 1 million (see Supplementary for more information).

### Experimental scheme

**Fig. 1:**
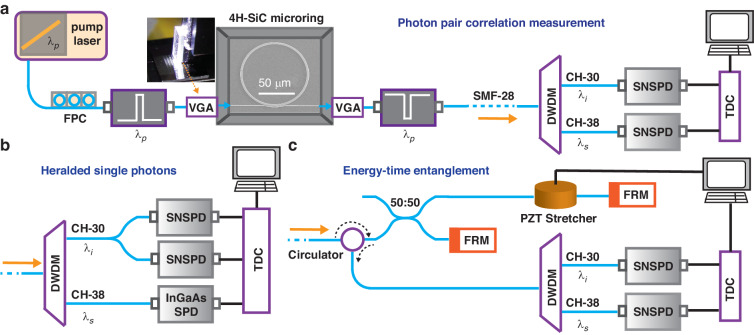
Experimental schematic. **a** Photon pair correlation measurement with the pump (*λ*_*p*_), signal (*λ*_*s*_) and idler (*λ*_*i*_) wavelengths chosen to be 1550.12 nm (CH34), 1546.92 nm (CH38), and 1553.33 nm (CH30), respectively. **b** Heralded single photon detection. **c** Energy-time entanglement measurement based on a folded Franson interferometer. FPC fiber polarization controller, VGA V-groove array, SNSPD superconducting nanowire single photon detector, DWDM dense wavelength-division multiplexing filter, TDC time to digital converter, PD photodetector and FRM Faraday rotator mirror

We summarize the major experimental setup for the device characterization in Fig. 1. First, the pump light, which is a continuous-wave telecom-wavelength tunable diode laser, goes through a narrow bandpass filter consisting of two cascaded 100-GHz dense wavelength division multiplexers (DWDMs) for selecting the laser line tuned into the ITU-34 channel (1550.12 nm). It is then coupled to the SiC chip via a grating-based coupler aligned to a fiber V-groove array (VGA), with approximately 5 dB of coupling loss on each side. To suppress the Raman noise generated in the fiber, the fiber length between the pump passband filter and the VGA input is reduced to 12 cm. The light coming out of the SiC chip is collected using another grating coupler before being sent to a pump rejection filter consisting of Bragg grating filters and 100-GHz DWDMs (>120 dB rejection). We then employ two similar DWDMs to filter the signal and idler photons into the ITU-38 (1546.92 nm) and ITU-30 (1553.33 nm) channels, respectively. The ITU-38 and ITU-30 channels are selected to leverage 100-GHz DWDM filters, as our device has a free spectral range of 400 GHz. However, we should be able to select other wavelengths as well. To ensure precise wavelength alignment to the ITU grids, the SiC chip is mounted on a temperature-controlled stage and the corresponding resonance positions are adjusted accordingly (see Supplementary for more information). Finally, the signal and idler photons are detected using two superconducting nanowire single-photon detectors (SNSPDs) with an approximate detection efficiency of 85% at 1550 nm and a dark count rate around 250 counts/s.

In the following, we will describe the three major experiments carried out in this work, i.e., the photon correlation measurement between the signal and idler (Fig. 1a), the heralded single photon detection (Fig. 1b), and the energy-time entangled photon pair generation (Fig. 1c).

### Photon correlation measurement

**Fig. 2:**
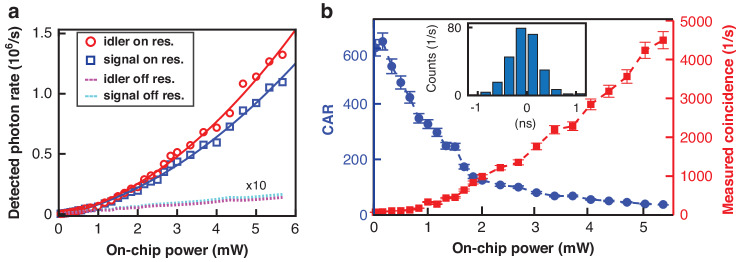
Photon pair generation in SiC. **a** Detected photon rate of the signal and idler as a function of on-chip pump power for both resonant (markers) and non-resonant pumping (dashed lines with the off-resonance number multiplied by a factor of 10 for better visibility). The on-resonance results are fitted by a quadratic curve in solid lines. **b** Measured coincidence-to-accidental ratio (CAR, left y-axis) and the detected coincidence count (right y-axis) as a function of on-chip pump powers with 0.24 ns of timing window. The inset shows a plot of coincidence histogram for 0.17 mW pump power corresponding to the measured CAR of 620. The temporal full width at half maxima (FWHM) width is approximately 0.68 ns

The signal and idler photons are strongly correlated since they are created at the same time while having to satisfy the energy and momentum conservation of the SFWM process. This strong correlation between the signal and idler photon pair forms the foundation of entanglement. Using the experimental schematic provided in Fig. 1a, we first characterize the detected rates of the signal and idler photons for both on-resonance and off-resonance pumping (Fig. 2a). As can be seen, the on-resonance pair generation rates exceed 1 × 10^6^ counts/s for an on-chip power near 5 mW, indicating a rate of > 10M photons/s on chip after accounting for all the insertion losses (approximately 10 dB) and detector efficiency ( ≈ 85%). The difference in the signal and idler flux is primarily attributed to their slightly different insertion losses. Both sets of data (represented by markers in Fig. 2a) are fitted by a quadratic curve with respect to the varying pump power, meeting the theoretical expectation reasonably well. On the other hand, the off-resonance photons are predominantly generated by the Raman scattering in fibers used in the experimental setup, as a similar level of noise is detected after bypassing the SiC chip (after accounting for the 10 dB insertion loss introduced by the SiC chip). In addition, both sets of off-resonance data (represented by dashed lines in Fig. 2a) display a linear dependence on the pump power, as is typical for Raman-induced noises.

To characterize the correlated photon pairs, we first obtain the histogram of the coincidence counts with a given size of time window (see for one example in the inset of Fig. 2b). The coincidence count (*C*_*c*_) is defined as the sum of the peak counts within the selected time bin, and the accidental count (*A*_*c*_) is obtained by averaging the events outside the coincidence peak using the same timing window. The CAR is then obtained by computing the ratio between the coincidence and accidental counts (i.e., *CAR* ≡ (*C*_*c*_ − *A*_*c*_)/*A*_*c*_). With this definition, we plot the measured CAR and the corresponding coincidences as a function of the input power in Fig. 2b for a time window of 0.24 ns. As can be seen, for 0.17 mW on-chip estimated pump power, we successfully measured a CAR of 620 without any background subtraction, which is a clear signature of correlated pair-detection events. The corresponding on-chip pair generation rate is estimated to be (9 ± 1) × 10^3^ pairs/s. With increased pump powers, the CAR value drops because of the increased multiple pair generation which is inherent in the SFWM process. Nevertheless, for an on-chip pump power near 5 mW, the measured CAR is still > 30 and the measured coincidence count is above 4000 counts/s. For a time window covering three standard deviations of the temporal width (which is ≈ 1.8 ns given that the signal/idler photons have a temporal span of 0.68 ns), the highest CAR is estimated to be approximately 125 at 0.17 mW of on-chip pump power, associated with an increase in the coincidence counts of up to more than 4 times (extra data available in Supplementary).

### Heralded single photon source

**Fig. 3:**
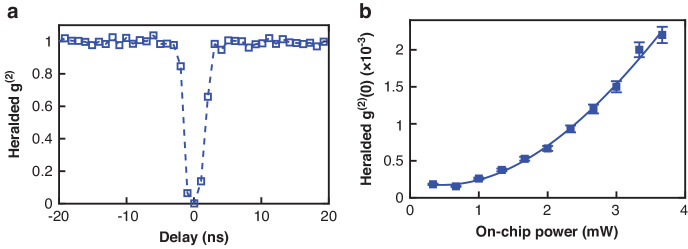
Heralded *g*^(2)^ measurement. **a** Heralded auto-correlation (*g*^(2)^(*τ*)) corresponding to an on-chip power of 2.6 mW. **b** Measured *g*^(2)^(0) (markers) as a function of the on-chip pump power, with the solid line representing the fitting curve

Following the photon correlation measurement, we proceed to characterize the single photon nature of the idler photon heralded by the signal photon detection (Fig. 1b): we add one InGaAs single photon detector to detect the signal photon (CH-38), which heralds the two SNSPD detectors for the detection of the idler photon (CH-30). The heralded auto-correlation histogram was obtained by analyzing the time-tag sequences of the three detectors. Antibunching is quantified by measuring the heralded auto-correlation (idler-idler) at the zero relative delay (*τ* = 0). Figure 3a shows one example of the measured *g*^(2)^(*τ*) corresponding to an estimated pump power of approximately 2.6 mW, which displays a minimum *g*^(2)^(0) around 10^−3^ indicating a clear antibunching. In Fig. 3b, we plot the heralded *g*^(2)^(0) as a function of estimated on-chip pump using 1 ns time bin window. Overall, the obtained *g*^(2)^(0) values are between 0.2 × 10^−3^ and 2.5 × 10^−3^, suggesting a high single-photon purity.

### Time-bin entanglement

**Fig. 4:**
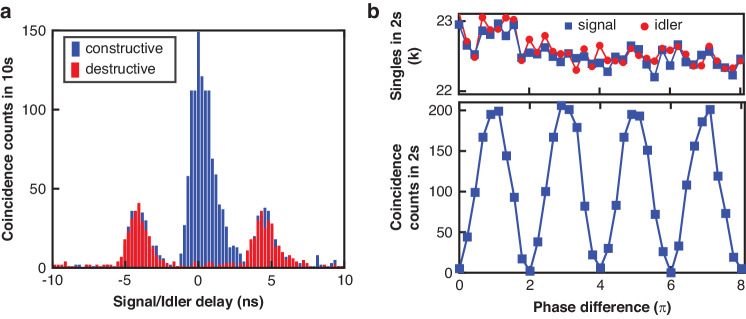
Two-photon interference of energy-time entanglement. **a** Histogram of constructive (blue) and destructive (red) interference of the energy-time entanglement measured in Fig. 1c. **b** Two-photon interference fringe obtained by varying the phase difference of the short-long paths in the unbalanced Franson interferometer

In the third part of the experiment, we move on to create energy-time entanglement using a folded Franson interferometer as described in Fig. 1c (the Faraday rotator mirror there doubles the optical path upon reflection). The coincidence histogram examples shown in Fig. 4a feature three peaks, which are characteristic of an unbalanced Mach-Zehnder interferometer (MZI). Essentially, the signal and idler photons can take either the long or the short path of the MZI with 50% probability. The two side peaks correspond to the cases that the signal and idler take two distinct paths (one takes the long path while the other takes the short path). However, if the photon pair travels along the same optical path, whether short or long, both events will contribute to the central peak indistinguishably and result in interference in the detection probability, whose amplitude depends on the relative phase of the interferometer. The two examples in Fig. 4a correspond to coincidence histogram of constructive and destructive interferences. By varying the phase of the interferometer, interference fringes of energy-time entangled photons can be measured as shown in Fig. 4b. The visibility is estimated to be (99.2 ± 0.4)% without background subtraction, which far exceeds the theoretical limit of $1/\sqrt{2}\approx 71 \%$ required to verify the entangled nature of photon pairs through test Bell’s inequalities^31^.

## Discussion

Our results, including a maximum CAR > 600 for an on-chip photon pair rate of (9 ± 1) × 10^3^ pairs/s and pump power of 0.17 mW, a heralded *g*^(2)^(0) on the order of 10^−3^, and a visibility of two-photon interference fringe exceeding 99%, unequivocally prove that the entangled photon source based on the SiC integrated platform could be a key resource for chip-scale quantum information processing. In addition, these results are comparable to those obtained from more mature nonlinear integrated photonic platforms such as silicon^2,32^, silicon nitride (Si_3_N_4_)^33–35^, aluminium gallium arsenide (AlGaAs)^36^, indium gallium phosphide (InGaP)^37^, and even lithium niobate^5,38,39^. In Table 1, we provide a selective comparison of SFWM-based entangled photon pair generation in various third-order nonlinear integrated photonic platforms. (Note that 4H-SiC used in this work is from Norstel (see Materials), which has a smaller Kerr nonlinear index *n*_2_ compared to Cree^30^.)

**Table 1:** Comparison of representative SFWM-based entangled photon pair generation in various *χ*^(3)^ integrated photonic platforms

References	Material platform	Kerr *n*_2_ (×10^−19^m^2^/W)	*Q* factor (million)	Pair rate (MHz)	Pump (*μ*W)	CAR	*g*^(2)^(0)	Visibility
Ma et.al.^32^	Si	≈30	≈0.1	1.1	≈59	≈532	≈0.005	≈98.9%
Fan et.al.^35^	Si_3_N_4_	≈2.5	≈1	0.024	≈250	1243 ± 469	≈0.014	≈99.4%
Steiner et.al.^36^	AlGaAs	≈260	≈1.2	1.0	≈6	2697 ± 260	≈0.004	≈97.1%
This work	4H-SiC	≈4.6	≈0.8	0.009	≈170	≈620	<0.001	≈99.2%

We believe that our study lends strong support to the competitiveness of the 4H-SiCOI platform for quantum applications. For example, the demonstrated entangled photon source can be readily deployed in a fiber-optic network for quantum communication. In addition, by aligning the wavelength of the idler photon to the zero-phonon line of various color centers found in SiC, we can create entanglement between the signal photon and the spin state. Such wavelength alignment can be done either through dispersion engineering or chip-scale frequency conversion^4,40^. All these possibilities point to a bright future of the SiC-based quantum photonics by integrating a multitude of chip-scale quantum photonic and electrical technologies with color centers for various applications.

## Materials and methods

The device fabrication starts with depositing 2-μm-thick PECVD oxide on an on-axis, semi-insulating 4H-SiC (Norstel AB) wafer, which is bonded to a silicon carrier and subsequently polished to a thickness of 800 nm (NGK Insulators)^19^. After dicing the 4-inch wafer into 1 cm × 1 cm chips, waveguides and resonators are patterned using e-beam lithography with FOx-16 as the resist. The pattern is subsequently transferred to the SiC layer with CHF_3_/O_2_ based dry etching, targeting for 650 nm SiC removal while leaving behind a 150-nm-thick pedestal layer. Finally, a layer of 1-μm-thick PECVD oxide is deposited on top of SiC for device encapsulation.

The on-chip grating coupler is designed for optimal coupling in the 1550 nm band for an SMF-28 fiber V-groove array with an eight-degree launch angle. The total insertion loss of the device, predominantly resulting from the coupling loss of the grating couplers, is estimated to be 10 dB. This signifies an approximate individual coupler loss of 5 dB.

The pump laser used in this work is a Toptica CTL1550 laser with a wavelength tuning range of 1460–1570 nm. This laser has a narrow linewidth of < 10 kHz, with its coherence length estimated to be larger than 100 m.

The DWDM filters employed in the experiment are single-channel filters with a 100 GHz channel spacing, exhibiting less than 1 dB insertion loss and a 13 dB Pass Channel with a 30 dB Reflect Channel. Specifically, we use the ITU-34 channel filters for filtering and rejecting the pump, while employing the ITU-38 and ITU-30 channels for selecting the signal and idler, respectively, ensuring direct compatibility with networking infrastructure. An additional fiber Bragg grating with 35 dB of isolation and a 1 nm bandwidth was used to further reject the pump wavelength.

The two SNSPDs detectors used in this experiment from Photon Spot are optimized for the C-band telecom wavelength, featuring an efficiency of ≈ 85% and a time jitter of ≈ 50 ns. An additional InGaAs single-photon detector from IDQ is employed for the *g*^(2)^ measurement, which has a quantum efficiency of around 25% and a time jitter of < 200 ns.

## Disclaimer

Certain commercial equipment, instruments, or materials are identified in this paper to foster understanding. Such identification does not imply recommendation or endorsement by the National Institute of Standards and Technology, nor does it imply that the materials or equipment identified are necessarily the best available for the purpose.

### Supplementary information

Supplementary Information for Entangled photon pair generation in an integrated SiC platform

## Data Availability

Data underlying the results presented in this paper are not publicly available at this time but may be obtained from the authors upon reasonable request.
