# Supplementary material for: Entangled photon pair generation in an integrated SiC platform
Source: Light Sci Appl. 2024 May 9;13:110. doi: 10.1038/s41377-024-01443-z (PMC11082171; doi:10.1038/s41377-024-01443-z)
Supplement: Supplementary file 1 — Supplementary Information for Entangled photon pair generation in an integrated SiC platform [file 41377_2024_1443_MOESM1_ESM.docx]

Supplementary Information for

Entangled photon pair generation in an integrated SiC platform

**Anouar Rahmouni*^1^, Ruixuan Wang^2^, Jingwei** **Li^2^, Xiao Tang^1^, Thomas Gerrits^1^,**

**Oliver Slattery^1^, Qing Li*****^2^, and Lijun Ma*****^1^**

*^1^ National Institute of Standards and Technology, 100 Bureau Dr, Gaithersburg, MD 20899, USA*

*^2^Department of Electrical and Computer Engineering, Carnegie Mellon University, Pittsburgh, PA 15213, USA*

**Corresponding authors:* [*anouar.rahmouni@nist.gov*](mailto:anouar.rahmouni@nist.gov)*,* [*qingli2@andrew.cmu.edu*](mailto:qingli2@andrew.cmu.edu) and [*lijun.ma@nist.gov*](mailto:lijun.ma@nist.gov)

Contents include:

1. **Linear characterization of SiC microrings and resonance wavelength tuning**
2. **CAR with time window covering three standard deviations**
3. **Characterization of Raman noise**
4. **Linear characterization of SiC microrings and resonance wavelength tuning**


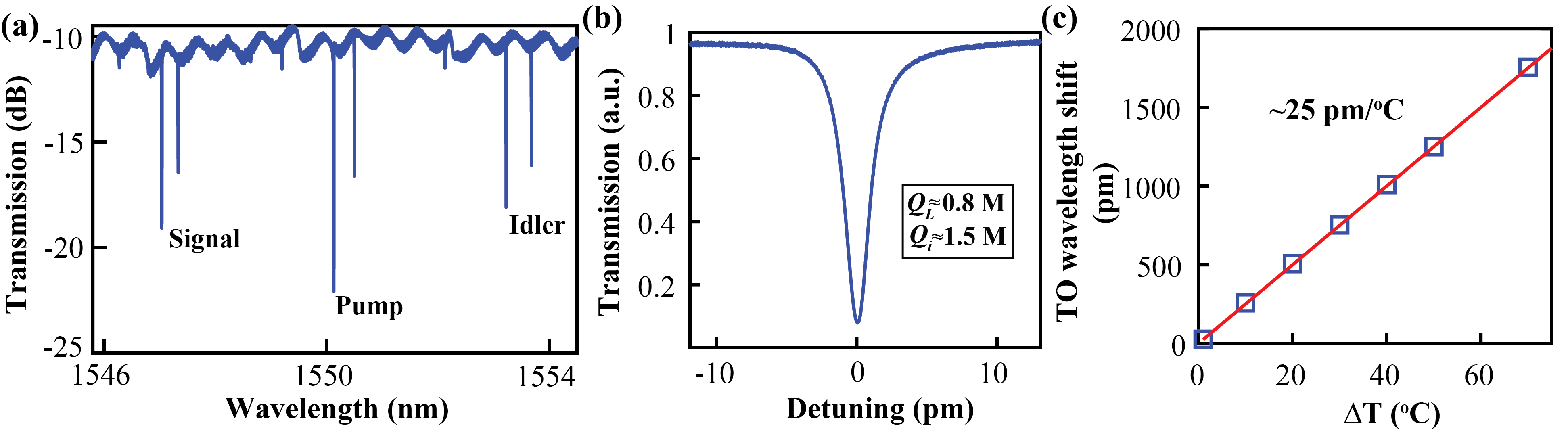


Figure S1: (a) Linear transmission scan of the 43-μm-raidus SiC microring used in this work. The ring width is 2 μm. The pump, signal and idler resonances belong to the same fundamental TM mode (TM_00_) families. (b) Close-up view of the pump resonance, showing an intrinsic quality Q_i_ of 1.5 million and a loaded quality Q_L_ of 0.8 million. (c) The pump, signal and idler resonance wavelengths are tuned together by varying the temperature of the sample stage. The blue markers are the measured wavelength shift, and the red solid line is a linear fit showing an approximate 25 pm/^o^C dependence.

©

Figure S1(a) shows absorption lines (resonances) corresponding to the fundamental TM modes that are evenly spaced, which are employed as signal, pump, and idler wavelengths. The other lines represent higher-order modes which are not used in the work.

1. **CAR with time window covering three standard deviations.**

Figure S2: Measured CAR and the corresponding coincidence count, without background substruction, as a function of estimated on-chip pump power where the timing window is chosen to cover ±3 standard deviations (∼2 ns).

1. **Characterization of Raman noise**

In this experiment, the off-resonance noise primarily originates from Raman scattering in the optical fibers employed in our setup. This assertion is supported by our observation that the noise is substantially reduced after shortening the fiber length between the optical filters and the SiC chip. In addition, we compared the noise level with and without the SiC chip while maintaining the same input pump power. After accounting for the insertion loss attributed to the SiC chip, we obtained nearly identical noise levels. Finally, the off-resonance photon counts (Fig. 2 in the main text) display a linear dependence on the pump power, consistent with the expected behavior of Raman-induced noise.

From the photon rate measured by the SNSPD in the off-resonance state ($N_{off\_res})$, the inferred Raman-induced noise photon ($N_{R})$ can be calculated by taking into account the insertion loss (${Loss}_{i})$ and the detection efficiency (𝜂) as:

$N_{R}=\frac{1}{\eta*{Loss}_{i}}*N_{off_{res}}.$ (1)

The Raman noise coefficient ($g_{r}$), which denotes the Raman-induced photons normalized by the pump power and the propagation length, can be computed using the following equation relating Raman-generated noise photon ($N_{R}$) to the pump power and optical fiber length as:

$N_{R}=g_{r}{*P}_{in}*\left( L_{1}*\left( 1-\int_{0}^{L1} e^{\alpha l}dl \right)+L_{2}*\left( 1-\int_{0}^{L2} e^{\alpha l}dl \right){*Loss}_{i} \right),$ (2)

where $P_{in}$ denotes the pump power at the input ($P_{out}={Loss}_{i}*P_{in}$), *L*_1_=12.5 cm is the fiber length prior to the SiC chip, *L*_2_=15 cm is the fiber length after the SiC chip, and $\alpha$ represents the propagation loss of the optical fiber. Given that our fiber length is short enough, we can neglect the fiber loss and obtain the Raman noise coefficient as:

$g_{r}\approx\frac{N_{R}}{P_{in}*L_{1}+P_{out}*L_{2}}$. (3)

Using the above formula, we plot the estimated Raman noise coefficient for various pump power levels in Fig. S3. Notably, the data consistently points to $g_{r}=800 \pm50 {mW}^{-1}{cm}^{-1}$(1-σ).

Figure S3: Estimated Raman noise coefficient from our experimental data for varied pump powers, resulting in a nominal value of $g_{R}=800 \pm50 {mW}^{-1}{cm}^{-1}$(1-σ).
